# Supplementary material for: Pathological Relationship between Intracellular Superoxide Metabolism and p53 Signaling in Mice
Source: Int J Mol Sci. 2021 Mar 29;22(7):3548. doi: 10.3390/ijms22073548 (PMC8037821; doi:10.3390/ijms22073548)
Supplement: Supplementary file 1 [file ijms-22-03548-s001.pdf]

Table S1. List of primers used for genotyping

Sod1 WT #1	AGC CCT GGT GCA GGA GTA TT
Sod1 WT #2	CCT ACC TTG TGT ATT GTC CCC A
Sod1 KO #1	TGT TCT CCT CTT CCT CAT CTC C
Sod1 KO #2	ACC CTT TCC AAA TCC TCA GC
p53 #1	GTT ATG CAT CCA TAC AGT ACA
p53 #2	ACA CCC AAC ACC ATA CCA TGT
p53 #3	CTT CCT CGT GCT TTA CGG TAT C
CAG-Cre #1	AGG TTC GTT CAC TCA TGG A
CAG-Cre #2	TCG ACC AGT TTA GTT ACC C
Sod2 flox #1	CGA GGG GCA TCT AGT GGA GAA
Sod2 flox #2	GAT GTC TGG ACA ACA TTA AGA AG
Sod2 flox #3	ACG ACC TGC AGC CAA GCT AGC TTG
p53 flox #1	AAG GGG TAT GAG GGA CAA GG
p53 flox #2	GAA GAC AGA AAA GGG GAG GG
